# DeepEOR: automated perioperative volumetric assessment of variable grade gliomas using deep learning

**DOI:** 10.1007/s00701-022-05446-w

**Published:** 2022-12-19

**Authors:** Olivier Zanier, Raffaele Da Mutten, Moira Vieli, Luca Regli, Carlo Serra, Victor E. Staartjes

**Affiliations:** Machine Intelligence in Clinical Neuroscience (MICN) Laboratory, Department of Neurosurgery, Clinical Neuroscience Center, University Hospital Zurich, University of Zurich, Frauenklinikstrasse 10, 8091 Zurich, Switzerland

**Keywords:** Glioma, Segmentation, Volume determination, Machine learning, Extent of resection, Neurosurgery

## Abstract

**Purpose:**

Volumetric assessments, such as extent of resection (EOR) or residual tumor volume, are essential criterions in glioma resection surgery. Our goal is to develop and validate segmentation machine learning models for pre- and postoperative magnetic resonance imaging scans, allowing us to assess the percentagewise tumor reduction after intracranial surgery for gliomas.

**Methods:**

For the development of the preoperative segmentation model (U-Net), MRI scans of 1053 patients from the Multimodal Brain Tumor Segmentation Challenge (BraTS) 2021 as well as from patients who underwent surgery at the University Hospital in Zurich were used. Subsequently, the model was evaluated on a holdout set containing 285 images from the same sources. The postoperative model was developed using 72 scans and validated on 45 scans obtained from the BraTS 2015 and Zurich dataset. Performance is evaluated using Dice Similarity score, Jaccard coefficient and Hausdorff 95%.

**Results:**

We were able to achieve an overall mean Dice Similarity Score of 0.59 and 0.29 on the pre- and postoperative holdout sets, respectively. Our algorithm managed to determine correct EOR in 44.1%.

**Conclusion:**

Although our models are not suitable for clinical use at this point, the possible applications are vast, going from automated lesion detection to disease progression evaluation. Precise determination of EOR is a challenging task, but we managed to show that deep learning can provide fast and objective estimates.

## Introduction

Glioblastomas (GBM), Oligodendrogliomas and Astrocytomas are the most common primary brain tumors [34, 49]. Magnetic resonance imaging (MRI) brain scans provide an essential modality for diagnosis, planning of therapeutic strategy and surveillance of such gliomas [45]. T1, T2, FLAIR and contrast T1 weighted are the standard imaging protocols used to fulfill these tasks [11, 43, 45]. Early postoperative MRI imaging is commonly carried out by most European centers, but still only a small fraction report a percentage wise reduction of tumor volume [43]. Extent of resection (EOR) achieved by maximum safe resection is a critical predictor for overall and disease-free survival as well as quality of life [6, 7, 22, 32, 33, 39], which is why early postoperative MRI imaging remains paramount [10, 23, 36]. However, manual segmentation of brain lesions is extremely laborious, somewhat imprecise and requires a certain degree of anatomical and pathological knowledge [5].

The latest convolutional neural networks (CNN), to which the UNet belongs, have been able to segment variable anatomical and pathological structures reliably and autonomously in a wide variety of medical images [18, 25, 30, 50]. Therefore, we believe that deep learning can be a valuable asset to improve patient care by facilitating volume calculations and streamlining EOR determination. We develop and validate deep learning models for segmentation of perioperative MRI scans, allowing volumetric assessment of variable grade gliomas.

MRI scans of gliomas can be divided into three subregions: enhancing tumor (ET), which corresponds to a region of relative hyperintensity in the contrast enhanced T1 sequence, non-enhancing tumor (NET), which is an area of relative hypointensity, often surrounded by ET in high grade gliomas and, lastly, edema (ED), which is best depicted by a hyperintensity in the FLAIR sequence. The union of these three regions is defined as whole tumor (WT) [11, 24]. An example of this partition is shown in Fig. 2.

## Methods

### Overview

To obtain a representative data set, first, an imaging registry of pre- and postoperative MRI scans from patients who underwent glioma resection surgery at the Department of Neurosurgery, University Hospital Zurich was hand-labeled. Using the said data together with additional data from the Multimodal Brain Tumor Segmentation Challenge 2015 and 2021 (BraTS), two ensemble learning model consisting of UNets were then trained and validated to segment ET, NET as well as WT on pre- and postoperative images.

### Ethical considerations

Patient data were treated according to the ethical standards of the Declaration of Helsinki and its amendments as approved by our institutional committee (Cantonal Ethics Committee Zürich, BASEC ID: 2021–01,147).

### Data sources

A database of 87 pre- and 92 postoperative images from patients that had variable grade gliomas resected at the Department of Neurosurgery of the University Hospital Zurich was hand-labeled by medical students, who had received prior expert teaching exclusively for this study (Zurich dataset).

For the preoperative model development MRI scans of 1053 patients from both the BraTS 2021 training set [2–4, 24] and Zurich were used. In a following step, the model was evaluated on a holdout set containing 285 images from the same sources. The BraTS 21 validation and testing data was not used in this study. The postoperative model was developed using 72 scans and validated on 45 scans, respectively obtained from both the BraTS 2015 [24, 57] and Zurich dataset. Detailed information on our dataset compositions can be found in Table 1.

**Table 1:** Data sources and allocation to study training and holdout sets. Cases from the Zurich dataset that underwent prior surgery are indicated in square brackets

Source	Study datasets			
datasets	Training		Holdout	
	Preoperative (*n* = 1053)	Postoperative (*n* = 72)	Preoperative (*n* = 285)	Postoperative (*n* = 45)
Zurich (USZ)	53 (5.0%)	58 (80.6%)	34 (11.9%)	34 (75.6%)
*LGG*	*20 (37.7%) [4 (7.5%)]*	*22 (37.9%)*	*12 (35.3%) [4 (11.8%)]*	*12 (35.3%)*
*HGG*	*33 (62.3%) [4 (7.5%)]*	*36 (62.1%)*	*22 (64.7%) [4 (11.8%)]*	*22 (64.7%)*
BraTS 2021	1000 (95.0%)	-	251 (88.1%)	-
BraTS 2015	-	14 (19.4%)	-	11 (24.4%)

*LGG*, low grade glioma; *HGG*, high grade glioma; *USZ*, University Hospital Zurich, *BraTS*, Brain Tumor Segmentation challenge

Operative procedures and preoperative assessments were conducted according to the current standards of care [42, 48]. Patients from the Zurich database were only selected, if all necessary 3 Tesla MRI protocols, namely T1, contrast enhanced T1 and FLAIR, were available in sufficient resolution and axial orientation. Preoperative imaging as well as postoperative scans no later than 3 months after surgery had to be available. Accordingly, patients with incomplete imaging as well as pediatric scans were excluded. However, a minority of patients included in this study already underwent prior brain tumor resection surgery but presented with recurrent lesions that required repeat surgery.

### Outcome measures

The segmentation models were trained to autonomously segment the glioma subregions ET, NET and WT on pre- and postoperative images of variable grade gliomas. The EOR was measured in an early postoperative MRI scan for 34 patients from the holdout set as the percentagewise reduction of tumor volume compared to baseline tumor volume on preoperative MRI.

### Metrics for segmentation evaluation

For evaluation of our deep learning–based glioma segmentations, we chose three metrics: The DICE similarity score and the Jaccard similarity coefficient, as overlap based metrics, and the Hausdorff metric, a distance-based calculation between two point sets [11]. As we used a two-dimensional UNet for image segmentations, consequently two-dimensional implementations were applied to calculate the metrics.

#### DICE Similarity Score (DSC, Sørensen–Dice coefficient, F1 Score)

The DSC considers the true positives, the false positives, and the false negatives. It is a measure of overlap being defined as twice the overlap between two areas A and B divided by their sum. It does not take true negatives into account [40, 56].

$$\mathrm{DSC}=\frac{2\left|\mathrm{A}\cap \mathrm{B}\right|}{\left|\mathrm{A}\left|+\right|\mathrm{B}\right|}$$

#### Jaccard Score (IoU, Intersection over Union Score)

The IoU is defined as the intersection over the union of two areas A and B [13]:

$$\mathrm{IoU}=\frac{\left|\mathrm A\cap\mathrm B\right|}{\left|\mathrm A\cup\mathrm B\right|}$$

The two metrics are very similar and positively correlated*.* Both range from zero — indicating no overlap — to one for perfect congruence.

*Hausdorff 95% distance (HD95):* The HD95 is defined as the 95^th^ percentile of the Hausdorff distance. The Hausdorff distance corresponds to the maximum distance from a border point of one area to the nearest point on the boundary of a second area, smaller values thus representing better performance. To eliminate the impact of outlying regions, the 95^th^ percentile of the Hausdorff distance is used [12, 14]. Note that HD95 scores were only calculated over regions that both contain information on the ground truth as well as algorithm segmentation concurrently.

### Model development and validation

As we take a clinical approach to deep learning and semantic segmentation, we primarily focus on basic procedures outlining their importance, rather than discussing every aspect in detail. All evaluations were executed using python 3.9.0 running Tensorflow 2.5.0 and keras 2.5.0 [1, 9, 46].

#### Pre-Processing

Medical imaging information is typically stored using the DICOM (Digital Imaging and Communications in Medicine) format. This, however, is not suitable for machine learning, thus making conversion to NIfTI (Neuroimaging Informatics Technology Initiative) filetype imperative [19]. In subsequent steps, the different MRI sequences need to be spatially aligned, the voxel size and image dimensions harmonized and lastly skull and soft tissue have to be removed to set the focus on brain parenchyma. We used a rigid transformation technique from SimpleITK for image coregistration [17] and MATLAB SPM12 fMRI tool for skull stripping. Skull stripping was carried out on T1 images and the brainmask was subsequently applied to all remaining sequences. These first few steps were not necessary for the images from the BraTS challenge datasets as they already fulfill the mentioned requirements. As a final step, the image intensity normalization was applied to each MRI sequence of each patient.

All steps described need to be carried out in a uniform manner when validating or using the models on new data.

#### Model development

**Fig. 1:**
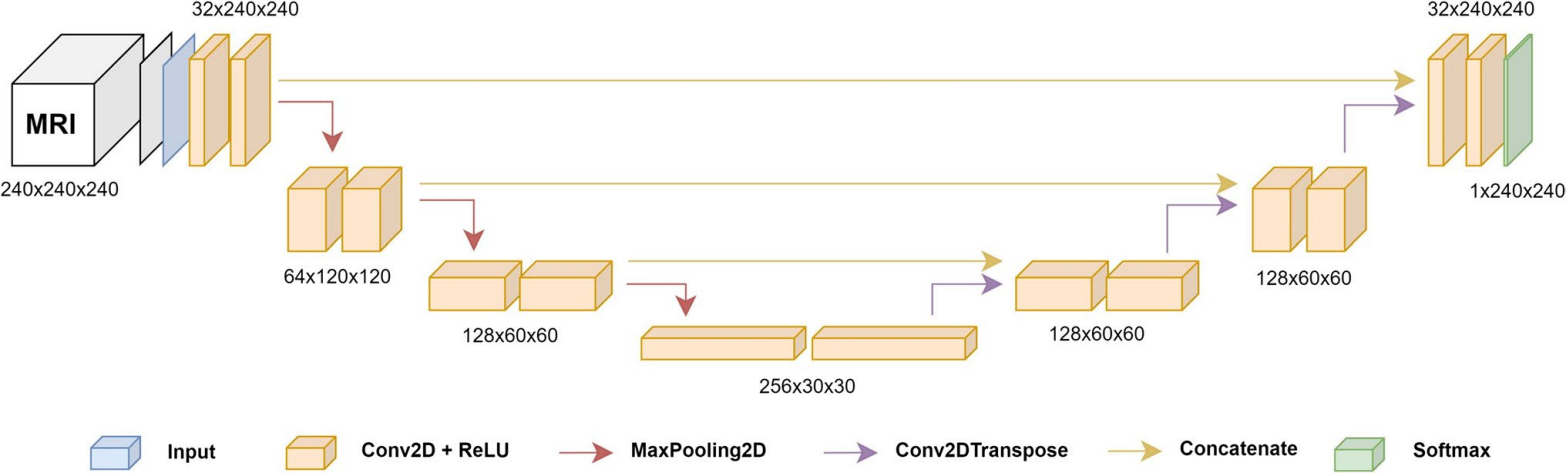
The baseline model architecture. A classic U-Net architecture is used, consisting of four levels with two consecutive sequences of convolution on the encoding as well as decoding part

The Python package Keras allows for a straightforward model training process by providing an efficient and user-friendly foundation for deep learning [9]. We used a basic 2D UNet structure [30] without any hyperparameter tuning during the model training process. Figure 1 illustrates a schematic of the model architecture. Although only two-dimensional, axial slices of the MRIs were used for 2D UNet model training and evaluation, the final segmentation results are three-dimensional. A fivefold cross validation [29] was used to train 5 models for each of the three tumor regions ET, NET, and WT which were subsequently ensembled. For ET and NET, the model was trained on T1 contrast-enhanced sequences while for WT, the FLAIR-weighted images were applied. The validation set was only used to observe the network’s performance during the training process and to assess its performance after training completion. Ranger optimizer, a combination of Rectified Adam [20] and Lookahead [55] optimizer, was used for stochastic optimization with binary cross entropy as loss function. The loss was computed batchwise using a batch size of 32. Each fold was trained for 40 epochs for preoperative models and 15 epochs for postoperative models with a learning rate of 0.001. To prevent overfitting, the below data augmentation techniques were applied:

- rotation range: ± 7 degrees
- zoom range: 90% (zoom in) and 110% (zoom out),
- horizontal and vertical image flip

For postoperative model training, we applied transfer learning, by retraining the preoperative models on postoperative data. This allowed us to transfer some of the knowledge already gained on the preoperative dataset into segmentation of postoperative imaging [44].

#### Post-processing

Outlying regions with a volume of less than 250 mm^3^ (0.25 ml) in preoperative and 50 mm^3^ (0.05 ml) in postoperative scans were removed.

#### Model evaluation

Training as well as testing performance were assessed using the above-mentioned DSC, IoU and HD95 metrics as well as volume correlation.

EOR was defined as the percentagewise volume reduction of ET + NET in postoperative MRI compared to baseline MRI before surgery. Algorithm segmentation deviation by more than 5% from ground truth EOR was considered incorrect. In contrast only values, whose deviation of the algorithm determined EOR from ground truth was less than 5%, were regarded as correct. EOR was evaluated on 34 patients from pre- and postoperative holdout set. It has to be noted that only patients that underwent surgery at the University Hospital Zurich were included in EOR evaluation, as the BraTS challenge datasets do not have reliable pre- and postoperative ground truth segmentations for the same patients. GTR was considered as EOR of 100% and performance of automated GTR determination was assessed using accuracy, sensitivity, specificity, positive predictive value, and negative predictive value metrics.

## Results

### Model performance

#### Segmentation task

Resampled and validation performance were assessed concordantly for the preoperative and postoperative models. The preoperative models achieved a mean DSC of 0.62 (± 0.30), 0.43 (± 0.34) and 0.73 (± 0.18) for ET, NET, and WT, respectively, on the holdout set. The Pearson coefficients for volume correlation amounted to 0.97 for ET and 0.37 for NET. WT volume correlation was 0.94.

Postoperative performance on the holdout set amounted to a mean DSC of 0.21 (± 0.23) and 0.07 (± 0.16) for ET and NET, as well as a DSC of 0.59 (± 0.24) for WT. Volume correlation was 0.89 for ET while the coefficient for NET amounted to 0.40. WT correlation reached 0.91.

**Fig. 2:**
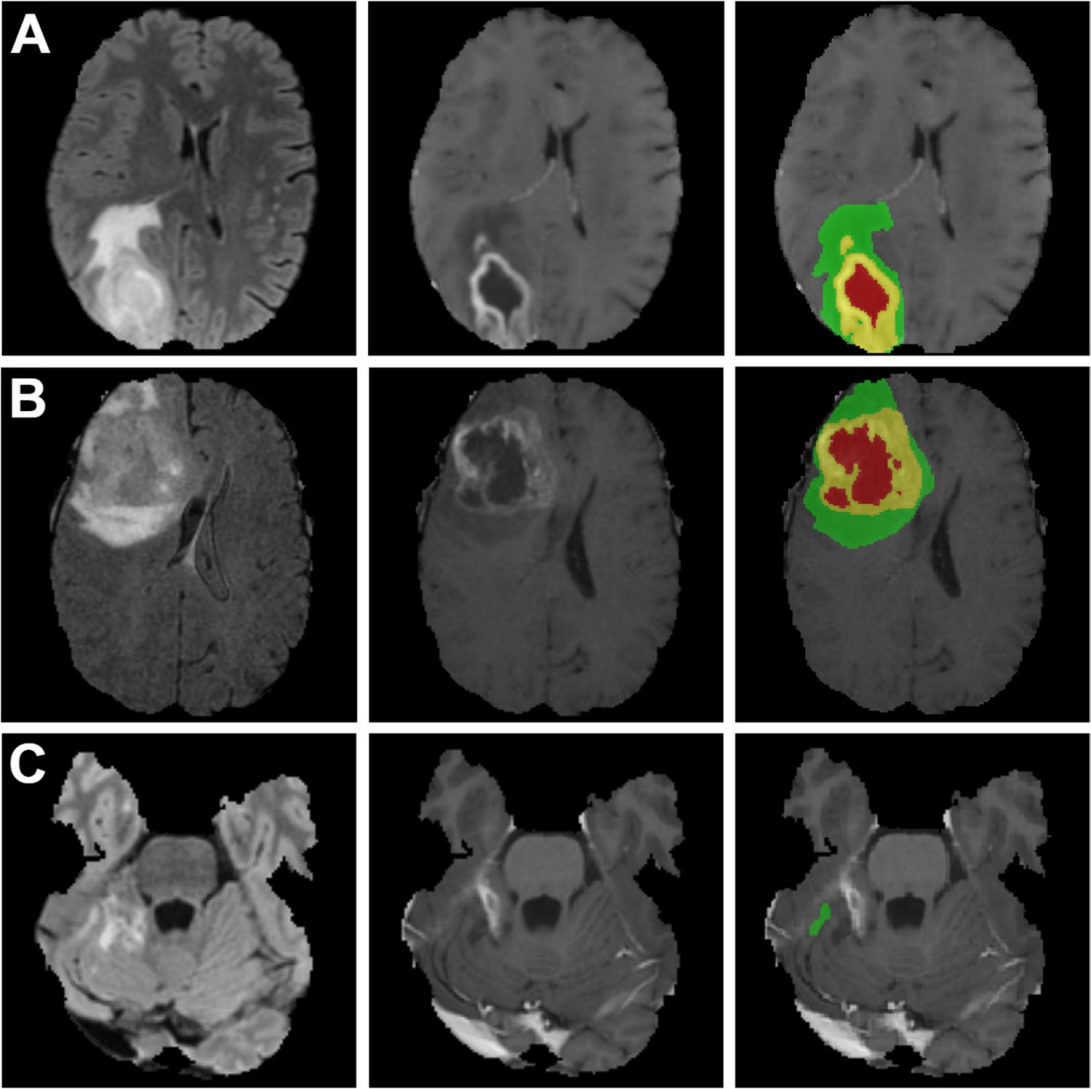
Preoperative holdout set results: Cases were differentiated as best, median or worst according to *patient wise mean DSC*. Within each row, the skull stripped FLAIR image is shown to the left, the T1 contrast enhanced image in the middle and an overlay with the generated segmentation to the right side. Edema is displayed in green, enhancing tumor in yellow and necrosis/non-enhancing tumor in red. Metrics are given as DSC: (A) **best:** ET 0.90, NET 0.97, WT 0.93, mean 0.93; (B) **median:** ET 0.67, NET 0.55, WT 0.82, mean 0.68; (C) **worst:** ET 0.0, NET 0.0, WT 0.0, mean 0.0

**Fig. 3:**
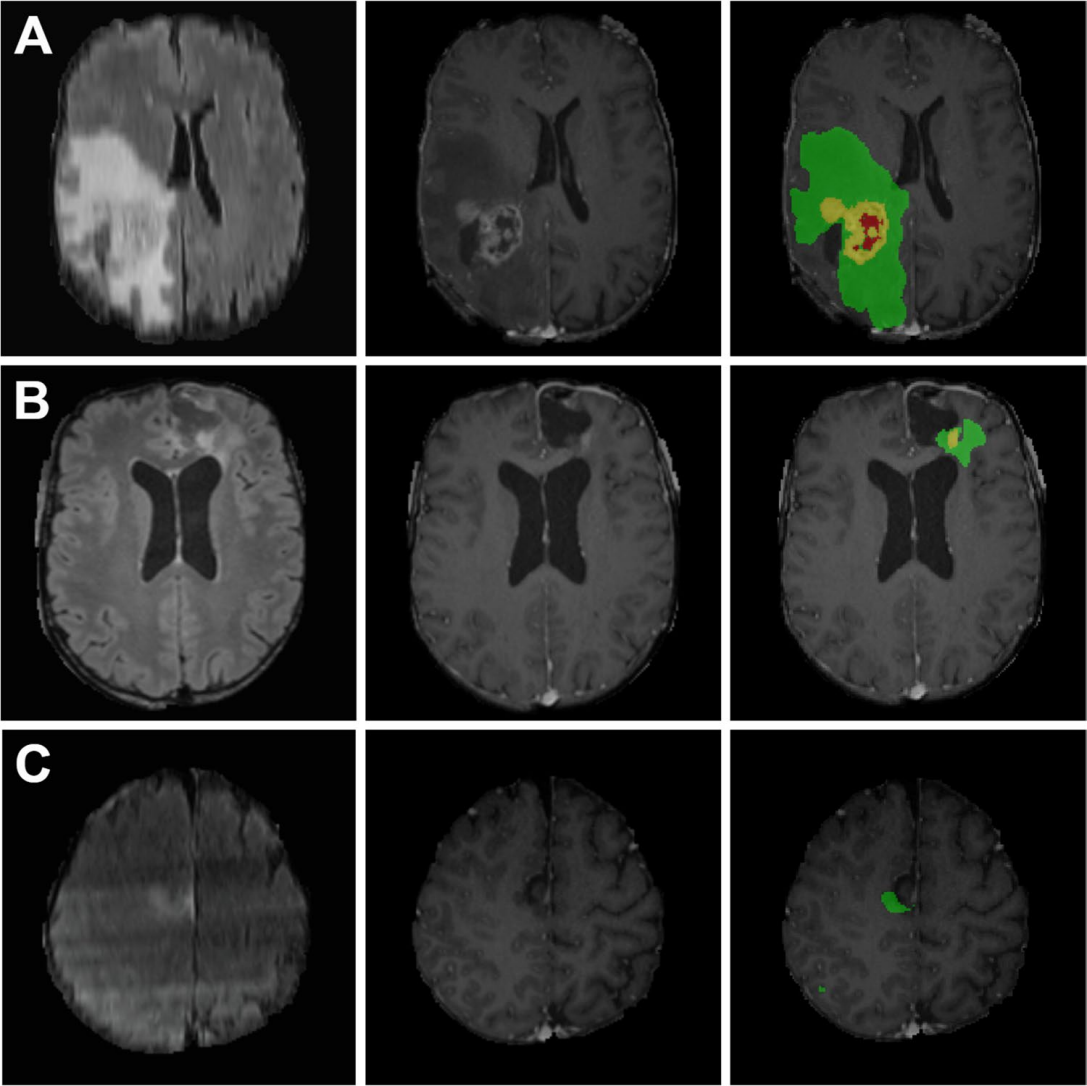
Postoperative holdout set results: Cases were selected as best, median and worst according to *patient wise mean DSC*. Within each row, the skull stripped FLAIR image is shown to the left, the T1 contrast enhanced image in the middle and an overlay with the algorithm generated segmentation to the right side. Edema is displayed in green, enhancing tumor in yellow and necrosis/non-enhancing tumor in red. Metrics are given as DSC: (A) **best:** ET 0.63, NET 0.50, WT 0.85, mean 0.66; (B) **median:** ET 0.12, NET 0.00, WT 0.74, mean 0.28; (C) **worst:** ET 0.0, NET 0.0, WT 0.05, mean 0.02

**Fig. 4:**
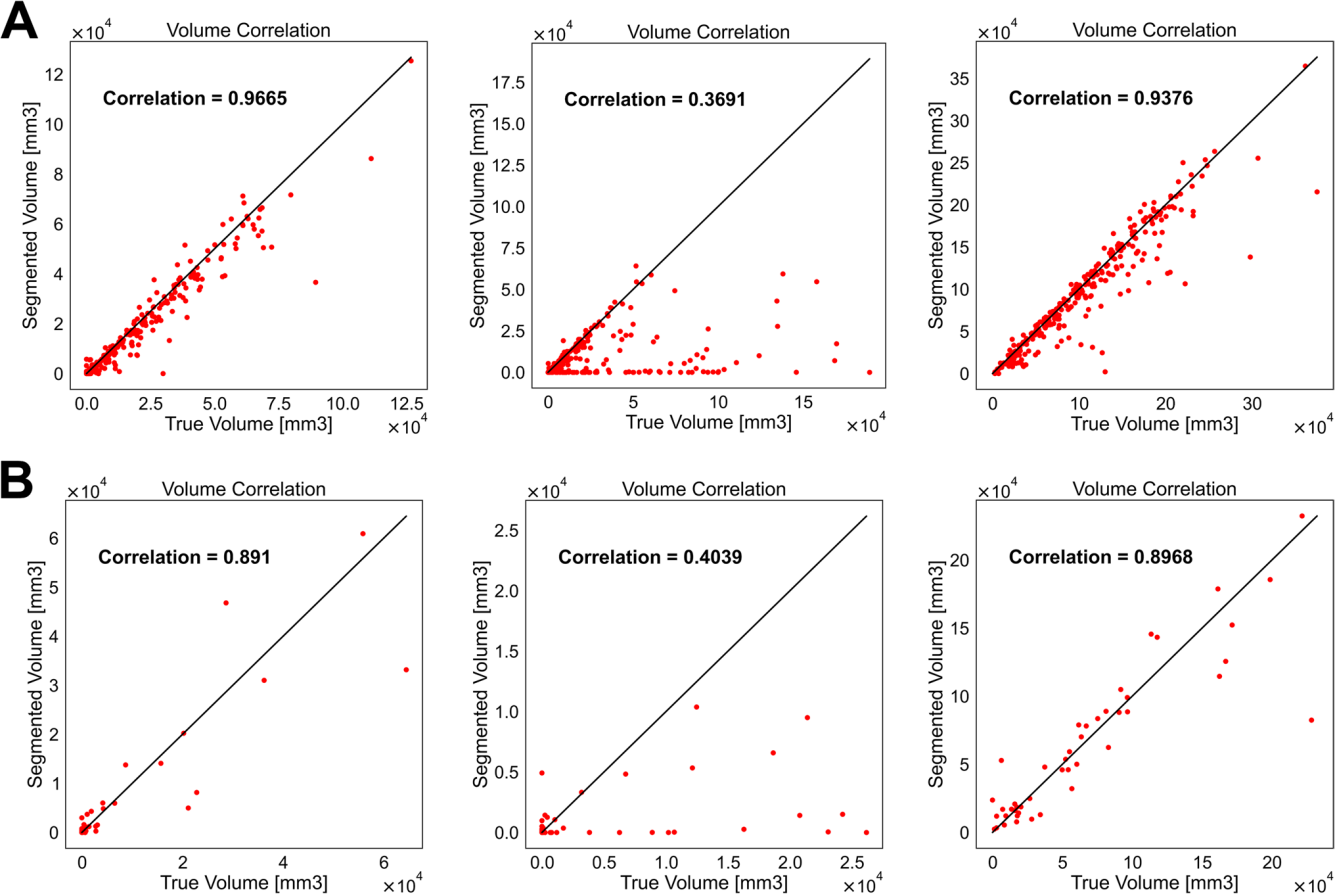
Volume Correlations on preoperative (A) and postoperative (B) holdout set. Within each row, ET volume correlation is shown to the left, the NET volume correlation in the middle and an WT volume correlation to the right. Pearson correlation coefficients are indicated inside the graph

Examples of our algorithm-based segmentations can be seen in Figs. 2 and 3. For a more detailed information on model performance, refer to Tables 2 and 3 as well as Fig. 4.

**Table 2:** Model performance on training and holdout set. Metrics are given as *cohort wise mean with median and interquartile range in brackets.* Note that while DSC and IoU are calculated over all slices that contain segmentations in either ground truth or algorithm segmentation, HD95 is only calculated over frames that contain segmentations in both ground truth and algorithm segmentation

	Thresh	DICE Similarity coefficient		Intersection over Union (Jaccard Score)		Hausdorff 95%	
Region		Training (*n* = 285)	Holdout (*n* = 1053)	Training (n = 1053)	Holdout (*n* = 285)	Training (*n* = 1053)	Holdout (*n* = 285)
Preoperative performance							
Enhancing tumor (ET)							
mean ± SD median (IQR)	0.5	0.73 ± 0.20 0.79 (0.68–0.86)	0.62 ± 0.30 0.75 (0.47–0.82)	0.66 ± 0.20 0.71 (0.58–0.80)	0.56 ± 0.28 0.67 (0.37–0.76)	4.19 ± 4.67 2.77 (1.85–4.64)	5.30 ± 5.48 3.25 (2.13–5.85)
Non enhancing tumor (NET)							
mean ± SD median (IQR)	0.4	0.64 ± 0.28 0.74 (0.49–0.85)	0.43 ± 0.34 0.51 (0.02–0.74)	0.57 ± 0.28 0.66 (0.41–0.79)	0.38 ± 0.32 0.40 (0.01–0.66)	5.87 ± 7.51 3.56 (2.00–7.01)	10.26 ± 11.14 5.94 (2.48–13.14)
Whole tumor (WT)							
mean ± SD median (IQR)	0.5	0.77 ± 0.15 0.80 (0.70–0.87)	0.73 ± 0.18 0.78 (0.67–0.85)	0.70 ± 0.16 0.74 (0.63–0.82)	0.67 ± 0.18 0.72 (0.60–0.80)	7.46 ± 6.43 5.41 (3.60–8.80)	8.07 ± 6.75 5.74 (3.59–9.99)
*Overall mean*		*0.71*	*0.59*	*0.65*	*0.53*	*5.84*	*7.88*
		Training (*n* = 72)	Holdout (*n* = 45)	Training (*n* = 72)	Holdout (*n* = 45)	Training (*n* = 72)	Holdout (*n* = 45)
Postoperative performance							
Enhancing tumor (ET)							
mean ± SD median (IQR)	0.1	0.18 ± 0.19 0.12 (0.0–0.31)	0.21 ± 0.23 0.13 (0.0–0.34)	0.14 ± 0.16 0.08 (0.0–0.23)	0.17 ± 0.19 0.10 (0.0–0.28)	11.56 ± 7.90 10.11 (5.51–16.06)	13.18 ± 9.02 10.14 (6.09–20.04)
Non enhancing tumor (NET)							
mean ± SD median (IQR)	0.25	0.02 ± 0.05 0.0 (0.0–0.02)	0.07 ± 0.16 0.0 (0.0–0.06)	0.01 ± 0.03 0.0 (0.0–0.01)	0.05 ± 0.13 0.0 (0.0–0.04)	24.66 ± 13.65 19.39 (15.63–29.48)	20.16 ± 10.49 18.84 (14.11–24.49)
Whole tumor (WT)							
mean ± SD median (IQR)	0.25	0.57 ± 0.22 0.63 (0.44–0.73)	0.59 ± 0.24 0.63 (0.43–0.80)	0.47 ± 0.20 0.52 (0.34–0.65)	0.50 ± 0.22 0.52 (0.33–0.69)	13.54 ± 8.17 12.22 (7.56–17.48)	14.18 ± 10.51 10.02 (6.34–18.13)
*Overall mean*		*0.26*	*0.29*	*0.21*	*0.24*	*16.57*	*15.84*

*SD*, standard deviation; *IQR*, interquartile range

**Table 3:** Model performance of low grade compared to high grade gliomas from 34 patients out of the Zurich part of the holdout set. Metrics are given as *cohort wise mean with median and interquartile range in brackets*

	Thresh	DICE Similarity coefficient		Intersection over Union (Jaccard Score)		Hausdorff 95%	
Region		Low grade	High grade	Low grade	High grade	Low grade	High grade
Preoperative performance							
Enhancing tumor (ET)							
mean ± SD median (IQR)	0.5	0.43 ± 0.39 0.42 (0.00–0.82)	0.74 ± 0.11 0.78 (0.71–0.82)	0.38 ± 0.35 0.32 (0.00–0.75)	0.64 ± 0.1 0.67 (0.60–0.70)	4.93 ± 4.29 3.5 (2.71–4.23)	3.29 ± 1.4 3.21 (2.17–3.77)
Non enhancing tumor (NET)							
mean ± SD median (IQR)	0.4	0.14 ± 0.24 0.00 (0.00–0.15)	0.58 ± 0.28 0.65 (0.55–0.76)	0.11 ± 0.19 0.00 (0.00–0.08)	0.49 ± 0.25 0.54 (0.44–0.66)	21.12 ± 11.78 23.1 (7.96–31.33)	6.91 ± 6.24 5.15 (2.00–8.67)
Whole Tumor (WT)							
mean ± SD median (IQR)	0.5	0.65 ± 0.23 0.72 (0.59–0.79)	0.80 ± 0.13 0.83 (0.79–0.87)	0.57 ± 0.21 0.72 (0.59–0.79)	0.72 ± 0.14 0.76 (0.69–0.81)	11.18 ± 9.17 8.09 (6.72–9.95)	7.89 ± 5.6 5.88 (4.71–8.29)
*Overall mean*		*0.41*	*0.71*	*0.36*	*0.62*	*12.41*	*6.03*
Postoperative performance							
Enhancing tumor (ET)							
mean ± SD median (IQR)	0.1	0.07 ± 0.14 0.00 (0.00–0.05)	0.21 ± 0.22 0.14 (0.00–0.33)	0.05 ± 0.11 0.00 (0.00–0.03)	0.17 ± 0.18 0.11 (0.00–0.26)	12.83 ± 7.63 12.89 (5.29–20.43)	12.73 ± 10.8 7.49 (5.23–18.50)
Non enhancing tumor (NET)							
mean ± SD median (IQR)	0.25	0.01 ± 0.03 0.00 (0.00–0.00)	0.11 ± 0.22 0.00 (0.00–0.07)	0.01 ± 0.02 0.00 (0.00–0.00)	0.08 ± 0.19 0.00 (0.00–0.04)	21.21 ± 1.70 21.21 (20.36–22.06)	15.52 ± 12.22 13.23 (8.00–18.39)
Whole Tumor (WT)							
mean ± SD median (IQR)	0.25	0.44 ± 0.26 0.39 (0.29–0.66)	0.67 ± 0.22 0.75 (0.57–0.83)	0.36 ± 0.23 0.31 (0.22–0.54)	0.58 ± 0.21 0.65 (0.47–0.73)	15.75 ± 10.29 12.89 (8.52–17.73)	12.38 ± 10.35 7.93 (6.12–15.40)
*Overall mean*		*0.18*	*0.35*	*0.14*	*0.28*	*17.60*	*13.54*

*SD*, standard deviation; *IQR*, interquartile range

#### EOR determination

**Fig. 5:**
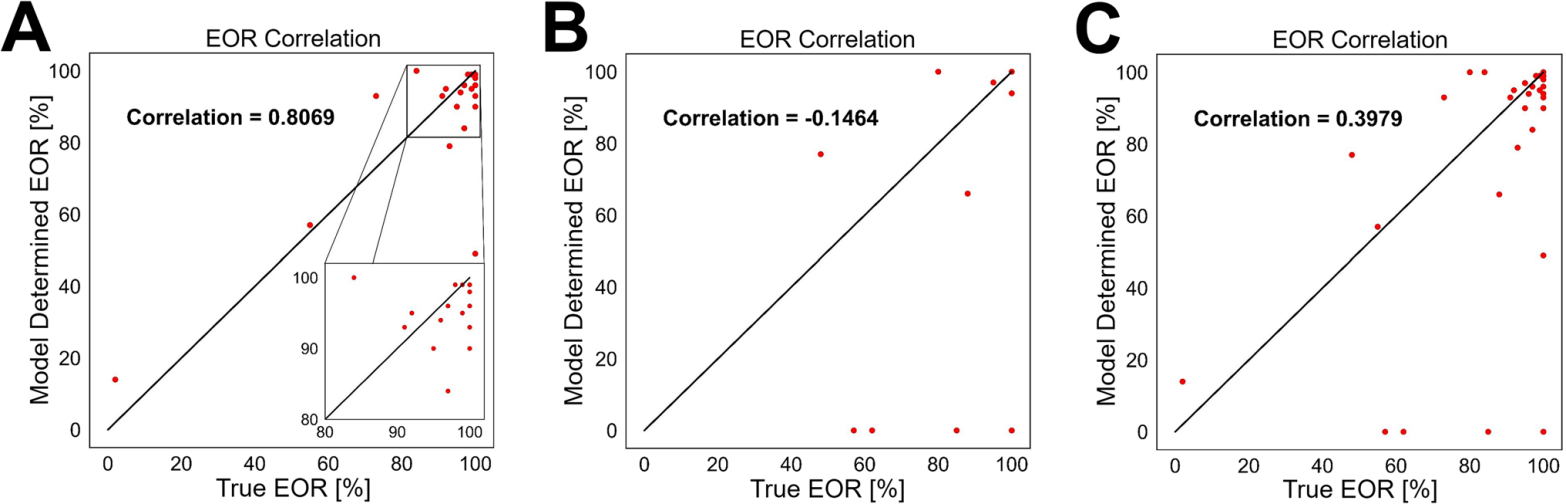
EOR correlation for 22 high grade gliomas only (A), for 12 low grade gliomas only (B) and over all 34 patients (C). Patients for whom the preoperative model did not segment any tumor were assigned a EOR of 0%. Pearson correlation is indicated in the graph

Our algorithm was able to measure correct EOR (deviation of less than 5% from ground truth EOR) in 15 out of 34 patients, which corresponds to 44.11% of patients (cf. Table 3). We managed to achieve a Pearson correlation of 0.40 on all 34 cases and 0.81 for 22 high grade glioma patients only (cf. Figure 5 and Table 5.

## Discussion

In this study, the feasibility of deep learning application in automated, volumetric lesion assessment as well as evaluation of EOR after surgical treatment of gliomas was investigated. With data from multiple registries ensemble learning models were trained and subsequently validated. The performance of our models was satisfactory on preoperative imaging and, given the difficulty of the task, acceptable on postoperative imaging. This showed that there is significant potential for clinical application of semantic segmentation algorithms. The objectivity and speed with which such models can assess volumetric information is unmatched. It is certain that further, systematic optimization of hyperparameters during model training and the use of pretrained segmentation models will further improve our model performance in the future [37].

There are a multitude of different architectures that are applied in medical imaging segmentation, the U-Net, on which we rely in this study, as well as different variations of convolutional neural networks (CNN) being among the most successful ones [30, 35]. Recently, Vision Transformers, have gained in popularity. Transformer models, which originally come from the field of natural language processing, are less computationally expensive and achieve performances comparable to state of the art CNNs [16, 26].

A main strength of our study is the inclusion of MRI scans from numerous different centers and scanners. Unlike Computer Tomography scans, intensities in MRI images are predisposed to significant statistical shift depending on different scanners and local protocols [51]. Including data from different centers therefore allows achieving a high level of generalizability, which is vital for projects intended to be applied in clinical practice. However, conversely this has a direct impact on model performance, potentially explaining the lack of better segmentation performance to some degree [51]. Additionally, the inclusion of some cases that underwent prior surgery in the Zurich dataset allows to extend applications of our models by making the dataset more comparable with “real world” data. As this might impede achieving higher segmentation performances, the effect of these secondary resection cases was compared to performance on primary resection cases only, as can be seen in Table 4, where no differences were observed. This is likely due to the low number of secondary resection cases included in this study.

**Table 4:** Model performance of primary resection cases only on the holdout set

	Thresh	DICE Similarity coefficient
Region		Holdout (*n* = 277)
Preoperative performance		
Enhancing tumor (ET)		
mean ± SD median (IQR)	0.5	0.62 ± 0.30 0.75 (0.47–0.82)
Non enhancing tumor (NET)		
mean ± SD median (IQR)	0.4	0.43 ± 0.34 0.50 (0.02–0.76)
Whole tumor (WT)		
mean ± SD median (IQR)	0.5	0.74 ± 0.17 0.79 (0.67–0.85)
*Overall mean*		*0.60*
Region		Holdout (*n* = 37)
Postoperative performance		
Enhancing tumor (ET)		
mean ± SD median (IQR)	0.1	0.21 ± 0.23 0.14 (0.00–0.32)
Non enhancing tumor (NET)		
mean ± SD median (IQR)	0.25	0.07 ± 0.17 0.00 (0.00–0.02)
Whole tumor (WT)		
mean ± SD median (IQR)	0.25	0.61 ± 0.23 0.66 (0.46–0.81)
*Overall mean*		*0.30*

**Table 5:** Volumetric model performance from holdout dataset compared to ground truth

Measurement (285 cases)	
Preoperative Volume	
Enhancing tumor Correlation (Pearson)	0.97
Non enhancing tumor Correlation (Pearson)	0.37
Whole tumor Correlation (Pearson)	0.94
Postoperative Volume	
Enhancing tumor Correlation (Pearson)	0.89
Non enhancing tumor Correlation (Pearson)	0.40
Measurement (34 cases)	
EOR [%]	
Correlation EOR (Pearson)	0.40
Difference in EOR [Median (IQR)]	6.5% (2.0–21.8%)
Difference in EOR [Mean ± SD]	36.9% ± 37.0%
Correlation EOR high grade only (Pearson)	0.81
Difference in EOR high grade only [Median (IQR)]	3.5% (1.3–11.5%)
Difference in EOR high grade only [Mean ± SD]	7.9% ± 11.0%
Correlation EOR low grade only (Pearson)	− 0.14
Difference in EOR low grade only [Median (IQR)]	29.0% (16.5–88.75%)
Difference in EOR low grade only [Mean ± SD]	46.25% ± 38.74%
GTR total	
Accuracy	0.59
Sensitivity	0.08
Specificity	0.86
PPV	0.25
NPV	0.63
GTR high grade	
Accuracy	0.64
Sensitivity	0.00
Specificity	0.88
GTR low grade	
Accuracy	0.58
Sensitivity	0.20
Specificity	0.86

Further, we counteracted overfitting by implementing image augmentation techniques and always carefully assessed its extent by comparing training against validation performance [38]. It cannot be excluded that the difference in performance between the training and holdout set of the preoperative NET model is partly due to overfitting, but apart from that, our results do not show major signs of overfitting.

We successfully applied transfer learning techniques which boosted performance of the postoperative models. Transfer learning makes it possible to relay some knowledge learned in a similar task into model training [44, 53]. By retraining the preoperative models on the postoperative data, we were able to partly compensate for low sample size and poor ground truth quality of the postoperative dataset.

A major challenge encountered during conducting this study was the evaluation of the postoperative model’s performance, especially for ET. This is due to multiple factors: First, the DSC and IoU punish false positives rigorously. As the residual-enhancing tumor areas for most subtotally resected high-grade gliomas are minuscule, even tiny false positive areas can have a huge impact on the final score [2]. However, it is much more probable to get false positives, as normal postoperative changes take up contrast agent. This represents a major challenge for all segmentation algorithms [5, 21]. An example can be seen in Fig. 3B; where the enhancing tumor is adequately labeled, but minor false positive areas in image slices that are not shown pull down the DSC for ET.

Secondly, there is a rather low interrater reliability for all postoperative ground truth segmentations [47]. This is commonly a known problem for postoperative imaging segmentations in general, as supervised learning techniques can only ever be as good as the “ground truth” data they have been trained on.

For the said reasons, it was a difficult task to derive reliable information on performance of postoperative models. We try to counteract this issue to some degree by supplementing volume correlation scatter plots, which can be seen in Fig. 4 and demonstrate a great comparability between algorithm results and ground truth segmentations for ET and WT.

Differences in interrater agreement of ground truth segmentations are also interesting topic for preoperative imaging: Since annotations of the BraTS and Zurich datasets are refined by a single annotator for each case and annotations are only approved by a second expert, it is not possible to provide any information specific for our data on the matter [2]. However, current literature suggests that preoperative interrater agreement is rather high [27, 47]. As discussed before, this is not the case for postoperative imaging.

Achieving a safe but high EOR is highly important for overall survival as well as disease-free survival, even if GTR is not reached [6, 7, 32, 33, 39]. Therefore, it is imminent to have the best possible understanding of the achieved EOR in order to deliver an accurate prognosis. However, segmentation models will always have a certain error rate. Thus, machine learning should never replace the careful study of imaging results. Rather, it should be seen as supplemental information available to physicians, aiming to facilitate, standardize and accelerate the processes involved in determining EOR.

There are studies with good results that used deep learning–based volumetric analysis of tumors to assess disease progression [28, 52], but to the best of the authors knowledge, no other studies have been conducted yet that aim at determining extent of resection on pre- and postoperative MRI imaging for brain tumors. A meta-analysis on the performance of machine learning algorithms by van Kempen et al. found the overall DSC to be 0.84 for preoperative glioma segmentations [15]. In a semi-automated approach for postoperative glioma, segmentation by Zeng et al. achieved an overall DSC of about 0.59 [15, 54].

Overall, the models developed in this study demonstrated adequate generalizability, performing similarly well on both test and training data. However, model performance depends on a multitude of variables, among them (sub)region of interest for segmentation, the imaging planes on which the model has been trained on, and the methods of segmentation metric calculation among others. These variables are handled inconsistently in current literature [41]. Using two-dimensional calculations for the metrics, as done in this study, leaves less room for error and impedes achieving higher scores compared to the respective three-dimensional implementations.

Besides automated EOR determination, our algorithm can be easily adapted to be able to autonomously detect lesions or evaluate tumor progression.

Segmentation of complex structures, like gliomas, remains a difficult task, but semantic segmentation algorithms can already provide adequate volumetric information in this study.

## Limitations

One limitation of our study is the relatively low sample size for postoperative model training. A decent surgical cohort of over 72 patients was included in training the models, which however still is a rather low sample size for deep learning [8]. Larger amounts of data and further hyperparameter tuning during model training would likely improve general model performance.

Furthermore, our algorithm was unable to segment NET of low-grade glioma in both pre- and postoperative models. This is also reflected in Table 3, where NET segmentation performance for low grade gliomas (DSC 0.14) is significantly lower than for high grade gliomas (DSC 0.58). The NET model, trained on T1 contrast enhanced sequences, often did not segment anything in low grade gliomas. This is due to the fact that the morphology of NET in glioblastomas differs fundamentally compared to low grade gliomas [47] and our models were not able to grasp this difference. Additionally, in T1 contrast–weighted images alone, the discrimination between edema and low-grade tumor can be extremely difficult, which further impedes accurate segmentation. However, even though overall performance for low grade gliomas was lower (cf. Table 3), the WT model, predicting on FLAIR sequences, was able to reliably segment low grade lesions with rather low discrepancy compared to the ground truths. This is essential, as it is common practice to carry out volumetric assessments of low-grade gliomas on FLAIR or T2 sequences [39].

As expert labels are very difficult to obtain, we mainly relied on postoperative ground truth segmentations from medical students and the BraTS 15 dataset for this study. However, the BraTS 15 postoperative ground truth labels are algorithm-based and therefore not on the qualitative level that would be desirable.

There are two further important drawbacks that are inherent when working with machine learning in general. First, all machine learning models are unable to reliably work with extreme cases that fall outside the range of the training data (extrapolation). If for example, a patient presents with glioma of the cerebellum, which is uncommon but realistic, a machine learning model trained on cerebral gliomas will not be able to segment it with the same reliability.

Second, the commonly known “black box” problem [31]: Especially with deep learning, one is often confronted with the inability to understand, why certain predictions have been made. By catering the algorithm with the required data, an accurate outcome can be derived. However, it remains unknown based on what aspects of the data these conclusions have been reached. While there are a lot of methods to make such models more transparent, most of them lack practical applicability.

## Conclusions

Precise determination of EOR after glioma resection surgery remains a challenging task, but deep learning offers potential in helping to provide faster and more objective estimates, which could aid in improving patient care. Especially for preoperative MRI imaging, the volumetric measurements correlate well with ground truth. Although our models are not ready for clinical application at present, we were able to deliver promising results developing and subsequently validating segmentation models for automatic volumetric measurements in patients that underwent surgery for variable grade gliomas.

## Data Availability

The data in support of our findings can be obtained upon reasonable request from the corresponding author.
